# Solution Structure and Peptide Binding of the PTB Domain from the AIDA1 Postsynaptic Signaling Scaffolding Protein

**DOI:** 10.1371/journal.pone.0065605

**Published:** 2013-06-14

**Authors:** Ekaterina Smirnova, Riya Shanbhag, Arwa Kurabi, Mehdi Mobli, Jamie J. Kwan, Logan W. Donaldson

**Affiliations:** 1 Department of Biology, York University, Toronto, Ontario, Canada; 2 Institute for Molecular Bioscience, University of Queensland, Brisbane, Australia; Torrey Pines Institute for Molecular Studies, United States of America

## Abstract

AIDA1 links persistent chemical signaling events occurring at the neuronal synapse with global changes in gene expression. Consistent with its role as a scaffolding protein, AIDA1 is composed of several protein-protein interaction domains. Here we report the NMR structure of the carboxy terminally located phosphotyrosine binding domain (PTB) that is common to all AIDA1 splice variants. A comprehensive survey of peptides identified a consensus sequence around an NxxY motif that is shared by a number of related neuronal signaling proteins. Using peptide arrays and fluorescence based assays, we determined that the AIDA1 PTB domain binds amyloid protein precursor (APP) in a similar manner to the X11/Mint PTB domain, albeit at reduced affinity (∼10 µM) that may allow AIDA1 to effectively sample APP, as well as other protein partners in a variety of cellular contexts.

## Introduction

Neurons receive chemical signals through a collection of over four hundred proteins that are organized into a network termed the postsynaptic density (PSD) [1]. AIDA1, a prominent member of the PSD, is edited into at least five isoforms, all of which contain two sterile alpha motif (SAM) domains and a PTB domain [2]. Together, these domains suggest a role for AIDA1 as a scaffolding molecule that collates proteins at the synapse through multiple protein-protein interactions. Mutations in AIDA1, consequently impair long term potentiation (LTP), a basic molecular requirement for learning and memory [3]. Owing to its role in many signaling processes, AIDA1 (located at chromosome 12q23.1) is also known as ANKS1B, ANKS2, cajalin-2 and EB-1.

AIDA1 derives its name from the ability to bind the carboxy terminal cytoplasmic region of amyloid precursor protein (APP), widely implicated in the development of Alzheimer’s disease. AIDA1 isoforms demonstrate differences in subcellular localization, affinity for APP and effect on the processing of APP to the Aβ40 nonpathologic fragment [2]. While AIDA1 is predominantly expressed in brain, a related protein, Odin (ANKS1A), with the same domain organization, is more ubiquitously expressed and serves as an adaptor modulating the signaling outcomes of epidermal derived growth factor receptor (EGFR), platelet derived growth factor receptor (PDGFR) and ephrin A8 receptor tyrosine kinase [4].

Previously, we determined the NMR structure of the AIDA1 SAM domain tandem and demonstrated that a nuclear localization signal was sequestered at the interface of the two domains [5]. In this report, we have continued a reductionist investigation of a potential AIDA1 SAM-SAM-PTB domain supramodule by determining the NMR structure of the PTB domain. The structure of the AIDA1 PTB domain and its ability to bind an NPxY motif in the amyloid precursor protein (APP) cytoplasmic region are similar to the postsynaptic signaling proteins APPL [6] and X11/Mint [7] and to a lesser extent, Fe65 [8]. Thus, the nature of signals arising from APP is likely dependent on the context specified by AIDA1 and the relative affinity of its competitors.

Our initial attempt to perform NMR structural studies on the AIDA1 PTB domain were impeded by poor solubility regardless of solution conditions chosen. Expression of the PTB domain from Odin (81% identity), presented an even worse case, as this protein fragment could not be refolded from inclusion bodies. A strategy that we pursued to improve the solubility of the AIDA1 PTB domain by involved the progressive substitution of aromatic amino acids that were predicted to be solvent exposed.

## Methods

### Cloning, Expression and Protein Purification

A gene fragment encoding the PTB domain (aa. 1043–1195) of human AIDA1b was PCR amplified with *NdeI* and *EcoRI* restriction sites and was subsequently inserted into pET28a (Novagen). The expressed protein contained an amino terminal 6xHis tag and intervening thrombin site. Other PTB domain fragments that lacked either the N-terminal 6xHis tag or the entire affinity tag along with 16 additional unstructured residues were also as insoluble as the fragment chosen for this study. To align the PTB domain described in this study with numerous AIDA1 isoforms, S1 in the PTB domain structure corresponds to S1045 in AIDA1b, the longest isoform. A one-liter fermentation in a minimal medium containing 1 g of ^15^NH_4_Cl and 4 g of ^13^C-glucose was sufficient to produce 5–10 mg of purified protein. Purification was achieved by Nickel-NTA affinity chromatography (Qiagen) and gel filtration chromatography on a S-100 HR 16/60 size exclusion column (GE Biosciences). Final buffer conditions were 20 mM Na-phosphate, pH 7.8, 0.15 M NaCl, 0.05% (w/v) NaN_3_. Five single aromatic-alanine substitutions (Y6A, F16A, F24A, Y70A and Y131A) were produced from pET28-AIDA1-PTB using a service provided by Genscript (Piscataway, NJ). A 6xHis tagged PTB domain variant containing all five substitutions (PTB5M) was produced by DNA2.0 (Menlo Park, CA) by direct gene synthesis in the expression vector, pJExpress401 (T5 promoter plus kanamycin resistance). A 6xHis-tagged, APP-peptide (GYENPTYKFFE) fused to the amino terminus of the AIDA1 PTB5M mutant with an intervening thrombin site was also synthesized by DNA2.0 in pJExpress401.

### Protein Solubility Assessment

Since the objective of the aromatic-alanine substitutions was to improve solubility for a structure determination, ^15^N-HSQC spectra were used qualitatively. From experience, the wild type AIDA1 PTB domain was soluble for a least one day at room temperate at a concentration of 0.15 mM thereby permitting experiments to be performed but to the extent of a structure determination. Each aromatic-alanine substitution mutant was concentrated to 0.15 mM, assessed by NMR and then concentrated until increased resonance line broadening was observed or there was apparent turbidity.

### CD Spectroscopy

Far UV circular dichroism (CD) spectra were acquired with a Jasco J-810 instrument at a protein concentration of 50 µM using a rectangular cell with a 0.1 cm path length. Spectra were recorded from 260–200 nm with a scan rate of 50 nm/min and a 1.0 nm bandwidth. A midpoint denaturation temperature (T_m_) was determined by heating samples from 20–90°C at 2°C/min and monitoring ellipticity at 222 nm.

### Protein Binding Studies

Fluorescein isothiocyanate (FITC) labeled peptides spanning portions of APP were produced and purified by CanPeptide (Montreal, QC) for fluorescence anisotropy based binding studies at 25°C using an Agilent Eclipse spectrophotometer equipped with a manual polarizer accessory. Buffer conditions were similar to those used for NMR spectroscopy. Measurements were made under identical conditions and averaged. Anisotropy was calculated from the relationship *(I_parallel_–GI_perp_)/(I_parallel_/2GI_perp_)* and normalized with the blank experiment. The equilibrium dissociation constant (K_D_) was calculated by direct fitting the titration curves with a standard two-state relationship using proFit 6.2 (Quantsoft).

### Peptide Array

A set of 12-mer peptides on a 150×100 mm cellulose membrane in a 10×30 array were synthesized using the SPOTS method [9] with an Intavis MultiPep instrument. A crude estimate of the peptide content in each spot was made by staining the array with Fast Green FCF. The array was probed with 1 µM of the solubility enhanced 6xHis-PTB5M mutant in PBST (3.2 mM sodium phosphate, 0.5 mM potassium phosphate, 1.3 mM KCl, 135 mM NaCl, 0.1% Tween-20, pH 7.4). Following blocking and washing with 5% skimmed milk and 2.5% bovine serum albumin (Bioshop Canada) in PBST, bound AIDA1 PTB was identified by incubating the array in a 1∶5000 dilution of horseradish peroxidase (HRP)-conjugated 6xHis monoclonal antibodies in PBST and developing with a chemiluminescent reagent (Santa Cruz Biotechnology). A complete table of peptides is provided in Supplementary Material as Table S1.

### NMR Spectroscopy

^15^N-edited HSQC spectra of the wild type PTB domain, mutants and protein-peptide complexes were acquired at 30°C on a Varian 600 MHz NMR spectrometer equipped with a salt tolerant cold probe. The use of a low protein concentration (0.10–0.15 mM) permitted assessment of all protein fragments regardless of intrinsic solubility. Chemical shift assignments on a uniformly ^15^N, ^13^C labeled sample of PTB5M at 0.8 mM were obtained using a conventional heteronuclear, triple-resonance strategy that incorporated non-uniform sampling for improved resolution and sensitivity. Backbone directed experiments: HNCACB, CBCA(CO)NH, HNCO, HNCACO, side chain directed experiments: H(C)(CO)NH, C(CO)NH, and ^13^C/^15^N-edited NOESY spectra were acquired on a Bruker Avance 900 MHz spectrometer equipped with a cold probe. Side chain HCCH-TOCSY, and aromatic HB(CBCG)CD, HB(CBCGCD)CE were acquired at 600 MHz. Protein solutions contained 10% D_2_O with the exception of the ^13^C-edited NOESY dataset in which the PTB5M sample was buffer exchanged into >95% D_2_O before data acquisition. Datasets were processed with NMRPipe [10] or the Rowland Toolkit [11] as required and interpreted with CCpNMR Analysis 2 [12]. Chemical shift assignments of PTB5M were deposited in the BMRB with the accession code 17934.

### Structure Determination

From an initial set of 500 structures calculated with CYANA 3, the top 20 structures were selected with no NOE violations >0.3 Å and no torsion angle violations <5°. This ensemble was then subjected to additional refinement in explicit solvent with a Python script (wrefine.py) supplied with XPLOR-NIH 2.30. The top 15 structures according to lowest refinement energy was deposited as an ensemble in the Protein Data Bank with the accession code 2M38. The ensemble was aligned using MOLMOL 2K1 [13].

### Structure Comparisons

Cα RMSDs and alignments between the AIDA1 PTB domain and related proteins were performed with PDBeFold [14].

### Peptide Docking Simulations

Starting from the AIDA1 PTB5M structure and APP peptide ligand placed in analogous position to that observed in the X11 PTB domain crystal structure [7], a two-stage docking simulation, at low resolution (200 structures) and then all-atoms high resolution (100 structures) was performed with FlexPepDock, part of the Rosetta 3.4 software package [15]. A low energy structure was selected for analysis.

## Results

**Figure 1:**
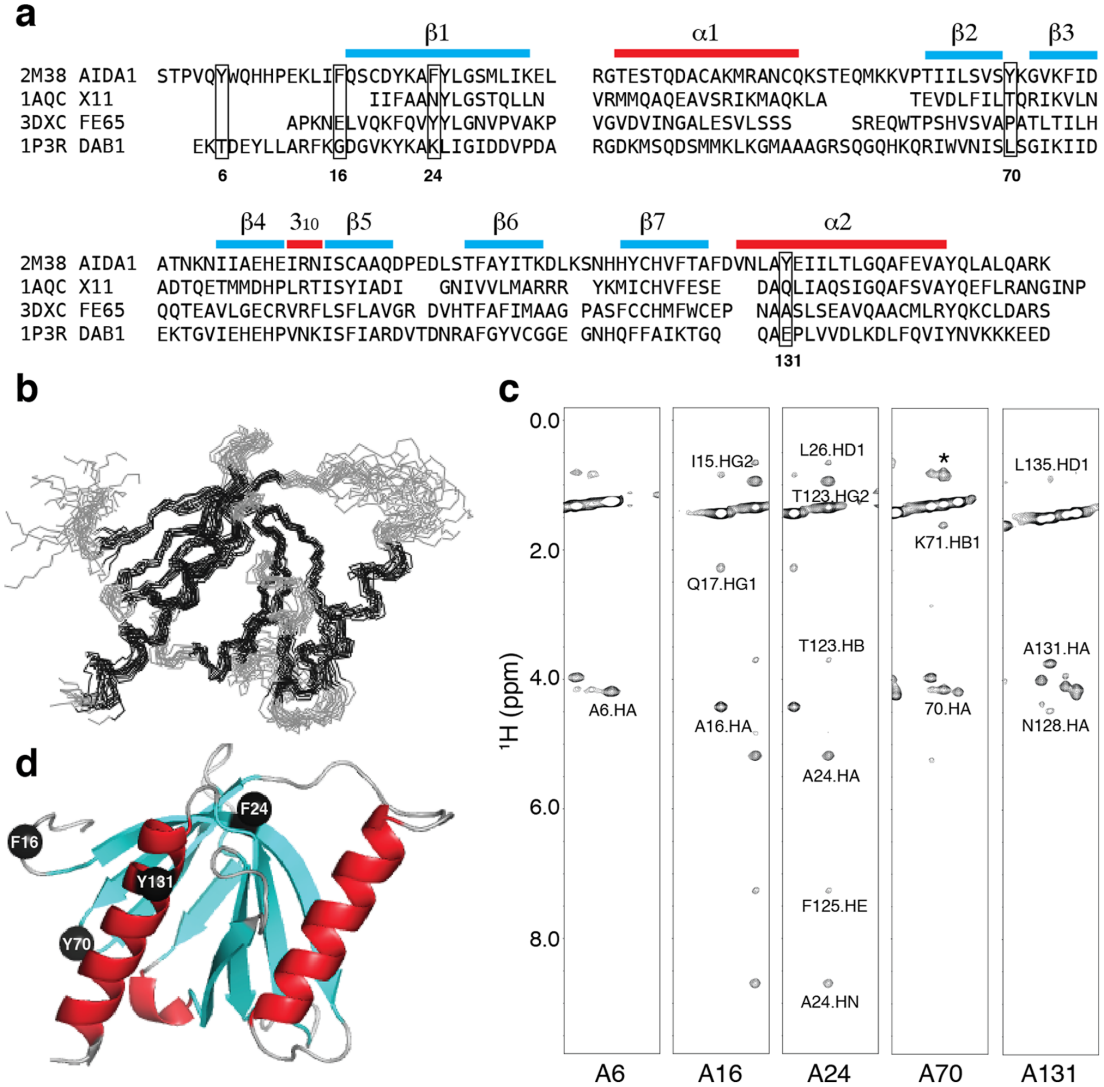
*(a)* Sequence alignment of the AIDA1 PTB domain against the APP binding proteins, Dab1 [25], X11 [17] and Fe65 [20]. Five aromatic amino acids selected for alanine substitution in AIDA1 PTB domain are boxed. *(b)* Backbone atom superposition of top15 structures according to lowest refinement energy. *(c)* Strip plots of a ^13^C-edited NOESY spectrum at the Cβ chemical shift of each alanine substituted in the PTB5M mutant. A asterisk denotes a resonance not associated with that strip. *(d)* A ribbon representation of the PTB5M model highlighting the positions of the alanine substitutions. Y6A is not shown in the figure as the first 14 amino acids are unstructured and were excluded from the structure calculation.

Prior to the structure determination, a molecular model of the AIDA-1 PTB domain was made with HOMA [16] using the crystal structure of the X11 PTB domain as the template [17]. Final refinement was performed with FOLDX [18]. The surface of the PTB model was scanned for exposed aromatics and compared to a sequence alignment consisting of the PTB domains from X11, Numb [19] and Fe65 [20]. Of the sixteen aromatics in the AIDA1 PTB domain, Y6, F16, F24, Y70, and Y131 were selected as candidates that were most likely to be surface-exposed (**Figure 1a**). While aliphatic amino acids could have been targeted as well, this decision would have added an additional layer of complexity. Thus, by selecting aromatic amino acids (Phe/Tyr/Trp considered equally), we were effectively sampling mutations under sparse conditions that still cover a wide range of surfaces.

As shown in **Table 1**, the calculated T_m_ of the mutants was comparable to the wild type PTB domain suggesting that the alanine substitutions did not destabilize the fold. Like the wild type PTB domain, the Y6A and Y131A single mutants could only be concentrated to 0.1 mM before precipitation was observed. The remaining single mutants – F16A, F24A and Y70A – could be concentrated up to 0.5 mM; however, HSQC spectra at these concentrations suffered from line broadening and missing resonances. In contrast, the PTB5M mutant was very soluble at 0.8 mM, with line widths that were comparable to the single mutants acquired at low concentration. Thus, we observed a synergistic effect when multiple aromatic amino acids were substituted with alanine.

**Table 1:** Solubilities and thermal denaturation midpoints of the AIDA PTB domain and alanine substitution mutants.

PTB domain	Tm (°C)	Solubility (mM)	Side chain exposure
wild type	62	0.10	N/A
Y6A	65	0.10	exposed
F16A	64	0.20	exposed
F24A	64	0.45	partially exposed
Y70A	64	0.45	exposed
Y131A	64	0.15	exposed
5M	64	0.80	N/A
APP-PTB	72	0.20	N/A
APP-PTB5M	73	0.80	N/A

The impact of the APP ligand on solubility was also investigated. The APP ligand was added exogenously, as a 17-mer peptide and endogenously, by appending the sequence to the amino terminus of the wild type PTB domain. Tethering a peptide ligand to a protein is a useful approach to to shift binding kinetics from biomolecular to unimolecular and ensure stoichiometric binding. In either case, addition of the APP ligand enhanced thermostability by 8°C but did not affect solubility. A structural determination of the APP-bound AIDA1 PTB domain was not pursued because there were fewer HN resonances a ^15^N-edited HSQC spectrum of the bound PTB domain (∼120) versus the free PTB domain (∼131) suggesting that ligand and binding cleft were severely line broadened beyond detection (**Figure S1**).

While the HSQC spectra of the Y6A, F16A, F24A, Y70A and Y131A PTB domains were all qualitatively similar in terms of chemical shifts and line widths, the F24A mutant spectrum was least similar to the other four mutant spectra under closer inspection suggesting that A24 could be making more structural contributions than the other alanine substitutions. Before the structure was determined (an ensemble of structures is shown in **Figure 1b**), we assessed the surface exposure of each aromatic-alanine substitution by examining the NOEs observed from the side chain methyl group. As shown in **Figure 1c**, only intramolecular and short range intermolecular NOEs were observed at A6, A16 and A70, suggesting that these methyl groups were significantly solvent-exposed. This was certainly the case for A6 as the chemical shift assignments indicated that the first 15 amino acids of the PTB domain were unstructured. Long-range NOEs were observed between the methyl group of A24 in β1 and the side chains of the adjacent β-strand (β7), specifically, the aromatic ring of F125 and the side chain of T123. The portion of the β-sheet in which substitution A24 resides was deemed to be resistant to hydrogen exchange as an NOE was observed between the methyl group of A24 and its own backbone amide despite the protein being dissolved in D_2_O. Taken together, these observations suggested that A24 was the least surface exposed of the five mutants chosen for the study. Once the structure determination was completed (a cartoon representation is shown in **Figure 1d**), these observations were confirmed and the F24A substitution appeared to be accommodated well. A PTB domain variant lacking the F24A substitution was not pursued because APP binding activity was unaffected.

The structure of the AIDA1 PTB5M mutant was aided substantially from data acquired at high field. A statistical summary is provided in **Table 2**. Overall, and as somewhat anticipated, the structure compares favorably to the other PTB domains that bind APP (**Table 3**). The PTB domain family can be divided into three major classes, namely Shc-like, IRS1-like and Dab-like [21], [22]: The AIDA1 PTB domain is a representative of the Dab-like class that binds non-phosphorylated-tyrosine peptides. While essentially complete chemical shift assignments were made, the α1-β2 loop spanning Q51-P62 remains unstructured and consequently dynamic due to a lack of long range NOEs observed throughout the region. The β6-β7 loop spanning K110-H116 also samples more conformations on average, supported by the observation that no resonance assignments could be attributed to N115.

**Table 2:** Restraints and Statistics for the Ensemble of 20 Structures.

**NOE restraints**	
Total	1127
Intraresidue (|*i* – *j*| = 0)	526
Sequential (|*i* – *j*| = 1)	183
Medium range (1< |*i* – *j*| <5)	125
Long range (|*i* – *j*| ≥5)	293
**Additional restraints**	
Hydrogen bond distance restraints (HN-N/HN-O)	58
Backbone angle torsional angle restraints	96
**RMS deviations from ideality** a	
Bonds (Å)	0.006±0.000
Angles (°)	0.557±0.021
Improper angles (°)	0.811±0.063
**RMS violations**	
NOE restraints	0.042±0.003
Dihedral angles (°)	0.051±0.067
**Ramachandran analysis for ordered residues** b	
Most favored regions	90.7%
Additional allowed regions	9.3%
Generously allowed regions	0.0%
Disallowed regions	0.0%
**RMSD to average coordinates for ordered residues**	
Backbone atoms (Å)	0.7
Heavy atoms (Å)	1.2

a As reported by XPLOR-NIH 2.33 using the standard protein force field.

b As reported by PROCHECK and selected by PSVS 1.4 for residues 17–51, 62–69, 72–114, 116–145.

**Table 3:** Structural similarity of the AIDA1 PTB domain to related PTB domains that also bind APP.

PDB	Protein	Source	RMSD	Aligned	Identity	Reference
2M38	AIDA1	NMR	0.0 Å	134 aa	100%	this study
1AQC	X11+ APP peptide	X-ray	1.4 Å	109 aa	26%	[17]
1P3R	DAB1	X-ray	1.6 Å	115 aa	27%	[25]
2ELA	APPL1	X-ray	1.7 Å	121 aa	16%	[23]
3DXC	Fe65+ APP peptide	X-ray	1.8 Å	120 aa	20%	[20]

**Figure 2:**
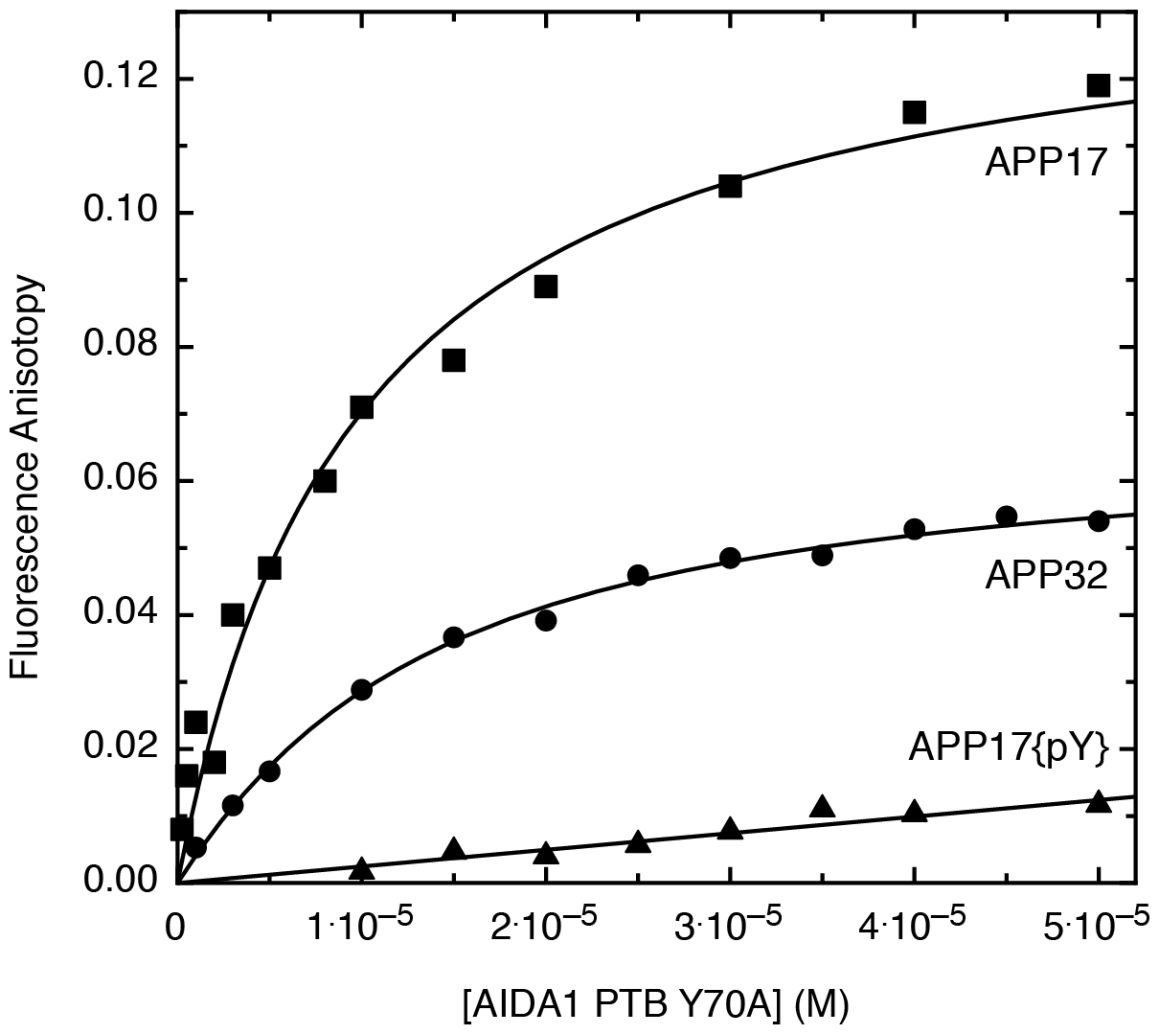
Titration of FITC-labeled APP peptides with a solubility enhanced mutant (Y70A) of the AIDA1 PTB domain. Binding was monitored by fluorescence anisotropy. Legend: APP17, a short X11-like binding site; APP32, a longer Fe65-like binding site; APP17{pY}, a short X11-like phosphopeptide. The peptide sequences are described in Table 4.

Structural and biochemical investigations of the Fe65 PTB2 domain demonstrated >100-fold difference in affinity between an 11 aa. minimal sequence (K_D_ = 100 µM) and an amino terminally extended 32 aa. (K_D_ = 0.2 µM) [8], [20]. One threonine (T668) in APP located in this extended region is susceptible to phosphorylation and acts as a switch that repartitions the *cis* and *trans* states of the adjacent proline (P669) that, in turn, affects the ability of Fe65 to engage its ligand. Titrations of long (APP32) and short (APP17) peptides showed no differences in binding affinity to the AIDA1 PTB domain suggesting that AIDA1, like many other PTB domains, binds an NPxY motif with a K_d_ of ∼10 µM (**Figure** **2** and **Table 4**). As predicted from the NMR structure, a semi-solubilizing Y70A single variant or the fully-solubilizing PTB5M variant had no affect on the affinity of the AIDA1 PTB domain to APP. An APP peptide bearing a phosphorylated Y687 did not bind the AIDA1 PTB domain providing further evidence for its inclusion in the Dab-like family.

**Table 4:** Affnities of APP-derived peptides for two solubility enhanced mutants of the AIDA1 PTB domain. ND: not done.

Ligand	Peptide sequence	K_d_ (µM)	
		PTBY70A	PTB5M
APP-32	ggDAAVTPEERHLSKMQQNGYENPTYKFFEQMQN	13.3±1.5 (n = 4)	14.8±1.0 (n = 4)
APP-17	ggQNGYENPTYKFFEQMQN	11.3±1.9 (n = 2)	11.5±1.4 (n = 2)
APP-17{pY}	ggQNGYENPT{pY}KFFEQMQN	ND	>1000(n = 1)

The K_D_ of the X11 PTB domain with a short APP peptide (14 aa., which is comparable to APP17 used in this study) is 0.3 µM, or over 100× stronger than the AIDA1 PTB domain [17]. From the perspective of the AIDA1 PTB domain, though, a lower affinity may not necessarily decrease its occupancy on APP relative to X11 and others, as the effective concentration of AIDA1 within the PSD is extremely high.

**Figure 3:**
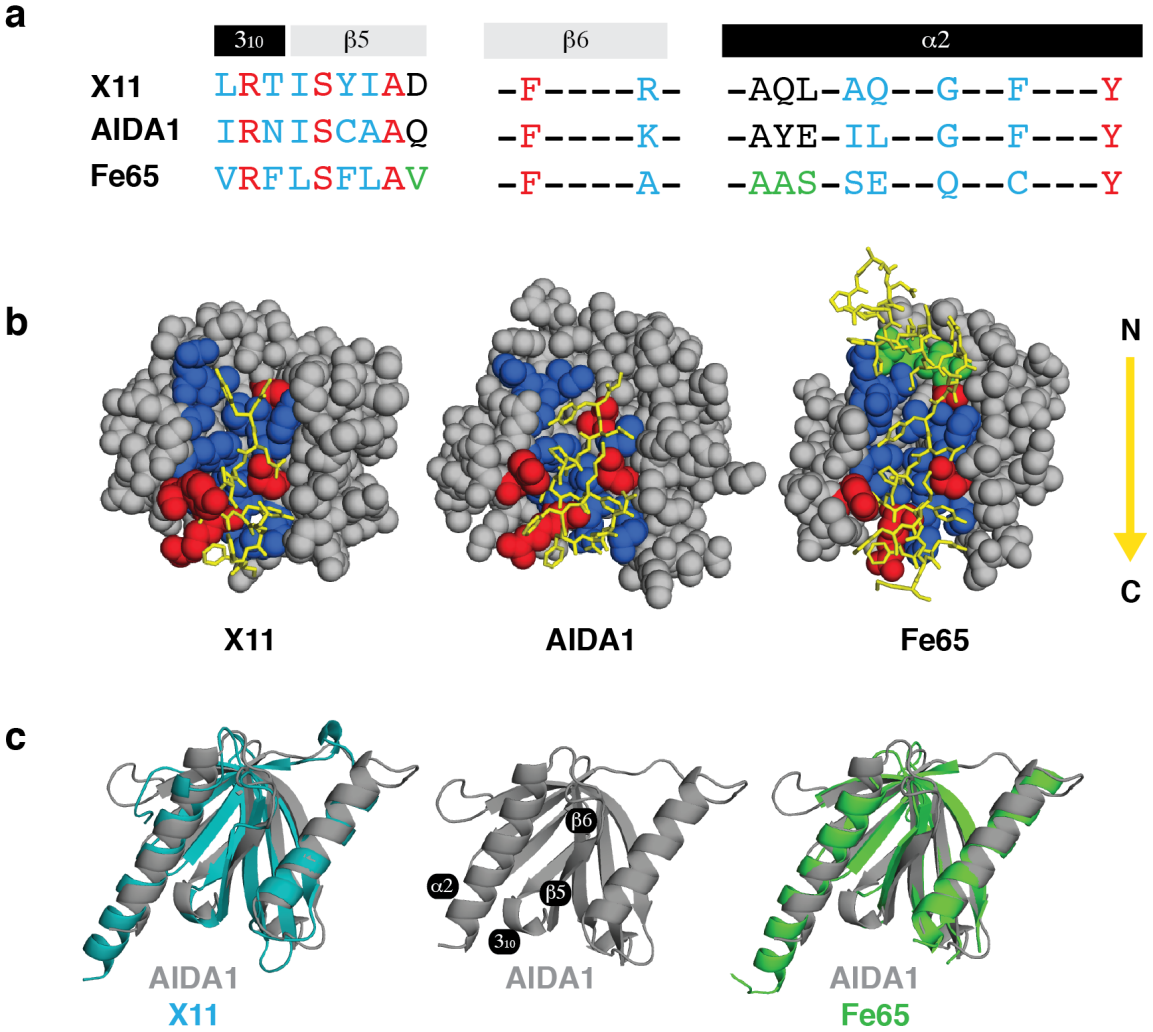
Interaction of a APP derived peptide (GYENPTYKFFE, shared among all) with the X11, Fe65 and AIDA1 PTB domains. *(a)* Sequence alignment of amino acids that contribute to the binding cleft; identity in red, homology in blue. The Fe65 PTB domain recognized a longer APP sequence, amino acids that extend its cleft are shown in green. *(b)* APP (yellow, in stick format, N-C direction follows the arrow) interacting with the X11/Fe65 as determined from their respective X-ray structures and with AIDA1 determined from a molecular docking simulation. (c) Backbone alignment of the AIDA1 (grey), X11 (cyan) and Fe65 (green) PTB domain in the same orientation as (b) with the binding cleft facing forward.

A comparison of the binding clefts of the AIDA1, X11 and Fe65 PTB domains is shown in **Figure 3**. The cleft of each PTB domain draws contributions from several secondary structures including a short, conserved 3_10_ helix, strands β5/β6 and helix α2. Surveying down the cleft, the first tyrosine of the APP GYENPTYKFFEQ peptide is positioned such that it is predominantly making contacts with G138 and F141 in α2 Analysis of the ensemble of peptides bound to the cleft from the Rosetta based docking simulation identifies an almost equal population of rotamers that place the tyrosine in an analogous position to what is depicted in the X11-PTB/APP complex. The alternative rotamer would contact I134 and L135 in α2 of the AIDA1 PTB domain. The second tyrosine of the APP GYENPTYKFFEQ peptide is contacted by a disparate set of amino acids among AIDA1, X11 and Fe65. In AIDA1, these residues are N91 in 3_10_ helix and K110 in β6. In X11 and Fe65, there is at least one supporting hydrophobic residue. The first of two consecutive phenylalanines in the APP GYENPTYKFFEQ peptide is supported by a tyrosine in all three PTB domain compared (Y145 in AIDA1).

**Figure 4:**
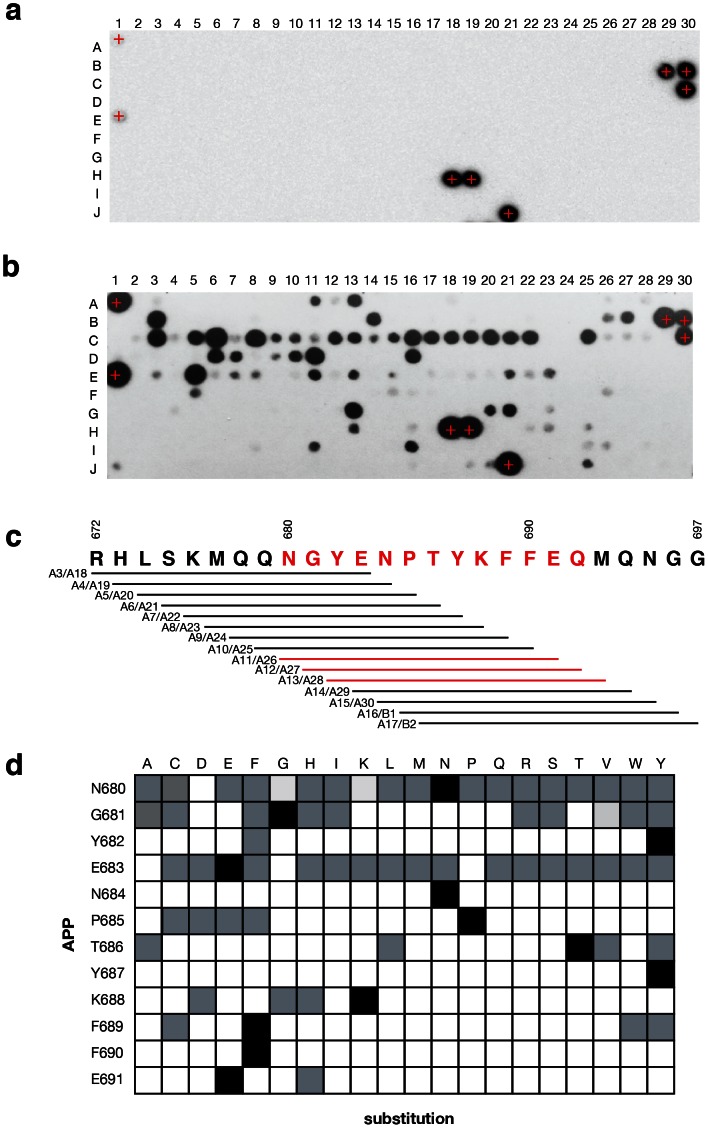
Amino acid preferences of the AIDA1 PTB domain for APP determined from a peptide array. A list of peptides on the array are provided in supplementary material. *(a)* The array probed with anti 6xHis mAb only. Positive control 6xHis peptides are identified by a **+**. *(b)* The array probed with 6xHis-AIDA1 PTB domain. *(c)* Sliding window peptide scan of 12-mers spanning aa. 672–697 of APP. Peptides are duplicated on the array; for example, at A3 and A18. Since peptide content per spot can vary, if a signal was observed at the exposure presented it was deemed to be interaction. *(d)* Results of a window scan across the APP C-terminal sequence and an exhaustive positional scan. Grey boxes indicate binding was observed, regardless of signal intensity.

A 12-mer SPOT peptide array (**Figures 4a and 4b**) was used to survey the amino acid preference of the AIDA1 PTB domain for APP and APP-like peptides. From an initial window scan of the APP carboxy terminal cytosolic region (**Figure 4c**), a minimal binding sequence of YENPTYKFFE was observed that is consistent with previously described peptide titrations and docking simulations. The minimal binding sequence was then used to exhaustively survey each position in the form of an ‘alphabet array’ (exhaustive amino acid substitutions at each position in the peptide). The results, summarized in **Figure 4d**, present a consensus sequence of YxNxΦYxΨFE where Φ is a hydrophobic amino acid and Ψ is an aromatic amino acid. Since the requirement for proline in the NPxY motif is not absolute, AIDA1 has the potential to sample NxxY motifs in receptors such as Ret that guides the development of neurons in the enteric nervous system [23]. If this is the case, a lower K_D_, and consequently a higher off-rate, would permit more ‘handshaking’ or sampling of potential protein partners to occur.

## Discussion

Our initial attempts at biochemical and structural studies of the AIDA1 PTB domain were precluded by poor solubility. As a result, we made five aromatic-alanine substitutions. While individual substitutions were helpful, it was the combination of all five substitutions that increased solubility to extent that an NMR structure determination was possible.

In addition to the solution structure of the AIDA1 PTB domain, we have determined that its affinity for unphosphorylated APP is moderate relative to similar APP binding proteins such as X11/Fe65 for which dissociation constants of <1 µM have been observed. This difference in affinity may be advantageous for AIDA1 to participate in signaling contexts beyond APP. From a peptide array study, we determined that the consensus sequence is less stringent NxxY versus NPxY for others in the same Dab-like class of PTB domains. Thus, at the neuronal synapse, AIDA1 could serve as a versatile collator and convenor of signaling events arising from the NMDA receptor, and possibly others.

Recent structural studies have revealed how the PTB domains of X11 [7] and Talin [24] are autoinhibited by flanking sequences. The AIDA1-APP interaction is antagonized by a short 26 aa. sequence specified by exon14 in some isoforms through an unknown mechanism [2]. The sequence itself, rich in hydrophobic amino acids, does not resemble the NPxY motif suggesting that regulation of the AIDA1 PTB domain may be occuring by non-competitive binding. Further structural and biochemical studies of AIDA1 may lead to selective modification of some neuronal signaling pathways while sparing others. Fine control of signaling pathways may be one strategy to improve preventive and anti-progression therapies of Alzheimer’s disease.

## Supporting Information

Figure S1 **A comparison of ^15^N-edited HSQC spectra from the** ***(a)*** **AIDA1 PTB5M protein and the** ***(b)*** **AIDA1 PTB5M protein with an APP binding sequence (GYENPTYKFFE) appended to the N-terminus along with a linker sequence (TLRPPNEATALQ) derived from the native AIDA1 protein.** Both protein concentrations are 0.8 mM.
(PDF)

Table S1 **A complete list of the 12-mer peptide sequences on the APP peptide array presented in** **Figure 4** **.**
(DOCX)
