# Supplementary material for: Solution Structure and Peptide Binding of the PTB Domain from the AIDA1 Postsynaptic Signaling Scaffolding Protein
Source: PLoS One. 2013 Jun 14;8(6):e65605. doi: 10.1371/journal.pone.0065605 (PMC3683042; doi:10.1371/journal.pone.0065605)

Supplementary Figure S1: A comparison of ^{15}N-edited HSQC spectra from the *(a)* AIDA1 PTB5M protein and the *(b)* AIDA1 PTB5M protein with an APP binding sequence (GYENPTY-KFFE) appended to the N-terminus along with a linker sequence (TLRPPNEATALQ) derived from the native AIDA1 protein. Both protein concentrations are 0.8 mM.

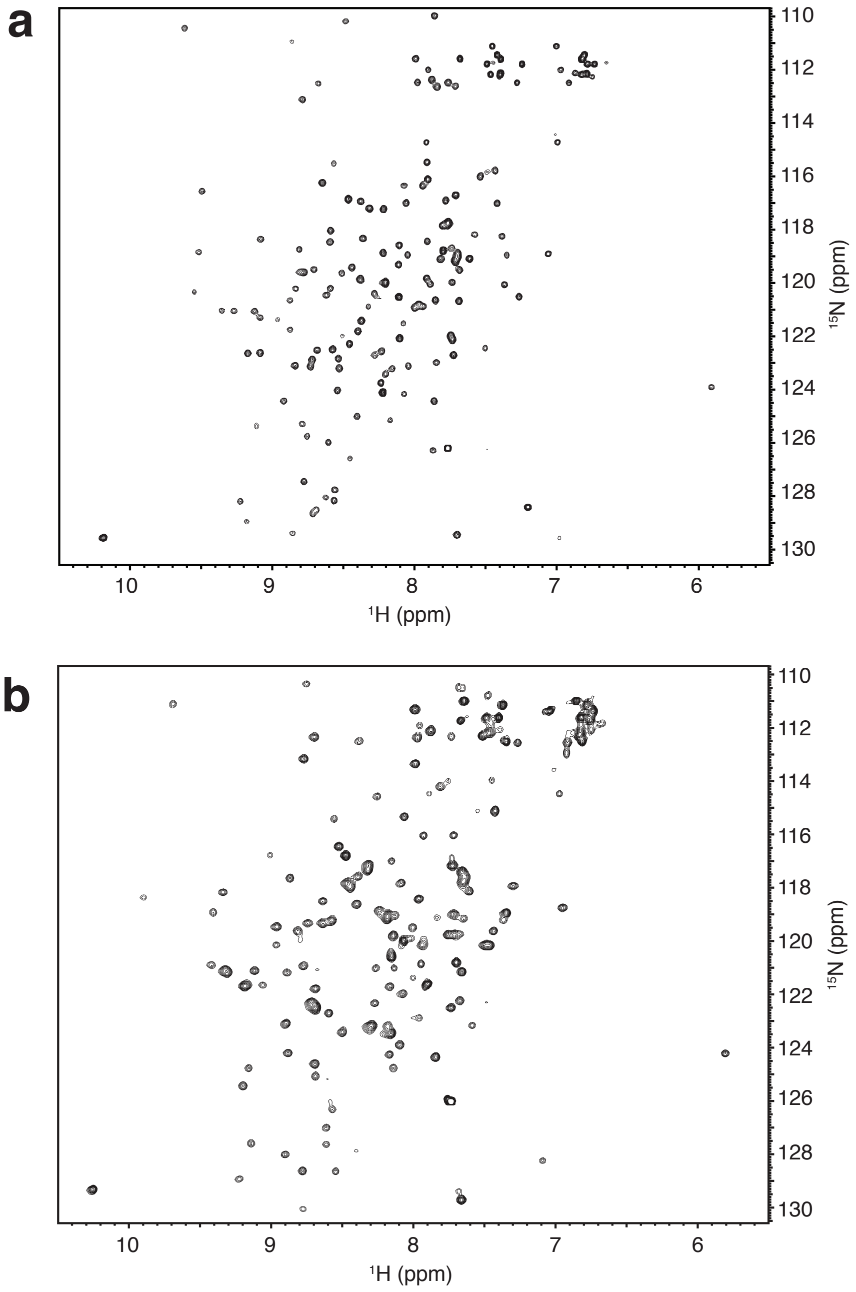

Supplement: Figure S1 — A comparison of 15N-edited HSQC spectra from the (a) AIDA1 PTB5M protein and the (b) AIDA1 PTB5M protein with an APP binding sequence (GYENPTYKFFE) appended to the N-terminus along with a linker sequence (TLRPPNEATALQ) derived from the native AIDA1 protein. Both protein concentrations are 0.8 mM. (PDF) [file pone.0065605.s001.pdf]
