# Supplementary material for: Solution Structure and Peptide Binding of the PTB Domain from the AIDA1 Postsynaptic Signaling Scaffolding Protein
Source: PLoS One. 2013 Jun 14;8(6):e65605. doi: 10.1371/journal.pone.0065605 (PMC3683042; doi:10.1371/journal.pone.0065605)
Supplement: Table S1 — A complete list of the 12-mer peptide sequences on the APP peptide array presented in Figure 4 . (DOCX) [file pone.0065605.s002.docx]

**Supplementary Table 1:** A complete list of 12-mer peptide sequences on the APP peptide array presented in Figure 4.

| 601 | A 1 | H-H-H-H-H-H-H-H-H-H-H-H |
| --- | --- | --- |
| 603 | A 3 | R-H-L-S-K-M-Q-Q-N-G-Y-E |
| 604 | A 4 | H-L-S-K-M-Q-Q-N-G-Y-E-N |
| 605 | A 5 | L-S-K-M-Q-Q-N-G-Y-E-N-P |
| 606 | A 6 | S-K-M-Q-Q-N-G-Y-E-N-P-T |
| 607 | A 7 | K-M-Q-Q-N-G-Y-E-N-P-T-Y |
| 608 | A 8 | M-Q-Q-N-G-Y-E-N-P-T-Y-K |
| 609 | A 9 | Q-Q-N-G-Y-E-N-P-T-Y-K-F |
| 610 | A10 | Q-N-G-Y-E-N-P-T-Y-K-F-F |
| 611 | A11 | N-G-Y-E-N-P-T-Y-K-F-F-E |
| 612 | A12 | G-Y-E-N-P-T-Y-K-F-F-E-Q |
| 613 | A13 | Y-E-N-P-T-Y-K-F-F-E-Q-M |
| 614 | A14 | E-N-P-T-Y-K-F-F-E-Q-M-Q |
| 615 | A15 | N-P-T-Y-K-F-F-E-Q-M-Q-N |
| 616 | A16 | P-T-Y-K-F-F-E-Q-M-Q-N-G |
| 617 | A17 | T-Y-K-F-F-E-Q-M-Q-N-G-G |
| 618 | A18 | R-H-L-S-K-M-Q-Q-N-G-Y-E |
| 619 | A19 | H-L-S-K-M-Q-Q-N-G-Y-E-N |
| 620 | A20 | L-S-K-M-Q-Q-N-G-Y-E-N-P |
| 621 | A21 | S-K-M-Q-Q-N-G-Y-E-N-P-T |
| 622 | A22 | K-M-Q-Q-N-G-Y-E-N-P-T-Y |
| 623 | A23 | M-Q-Q-N-G-Y-E-N-P-T-Y-K |
| 624 | A24 | Q-Q-N-G-Y-E-N-P-T-Y-K-F |
| 625 | A25 | Q-N-G-Y-E-N-P-T-Y-K-F-F |
| 626 | A26 | N-G-Y-E-N-P-T-Y-K-F-F-E |
| 627 | A27 | G-Y-E-N-P-T-Y-K-F-F-E-Q |
| 628 | A28 | Y-E-N-P-T-Y-K-F-F-E-Q-M |
| 629 | A29 | E-N-P-T-Y-K-F-F-E-Q-M-Q |
| 630 | A30 | N-P-T-Y-K-F-F-E-Q-M-Q-N |
| 631 | B 1 | P-T-Y-K-F-F-E-Q-M-Q-N-G |
| 632 | B 2 | T-Y-K-F-F-E-Q-M-Q-N-G-G |
| 633 | B 3 | A-A-Y-E-N-P-T-Y-K-F-F-E |
| 634 | B 4 | N-A-A-E-N-P-T-Y-K-F-F-E |
| 635 | B 5 | N-G-A-A-N-P-T-Y-K-F-F-E |
| 636 | B 6 | N-G-Y-A-A-P-T-Y-K-F-F-E |
| 637 | B 7 | N-G-Y-E-A-A-T-Y-K-F-F-E |
| 638 | B 8 | N-G-Y-E-N-A-A-Y-K-F-F-E |
| 639 | B 9 | N-G-Y-E-N-P-A-A-K-F-F-E |
| 640 | B10 | N-G-Y-E-N-P-T-A-A-F-F-E |
| 641 | B11 | N-G-Y-E-N-P-T-Y-A-A-F-E |
| 642 | B12 | N-G-Y-E-N-P-T-Y-K-A-A-E |
| 643 | B13 | N-G-Y-E-N-P-T-Y-K-F-A-A |
| 644 | B14 | Q-G-Y-E-N-P-T-Y-K-F-F-E |
| 645 | B15 | N-Q-Y-E-N-P-T-Y-K-F-F-E |
| 646 | B16 | N-G-Q-E-N-P-T-Y-K-F-F-E |
| 647 | B17 | N-G-Y-Q-N-P-T-Y-K-F-F-E |
| 648 | B18 | N-G-Y-E-Q-P-T-Y-K-F-F-E |
| 649 | B19 | N-G-Y-E-N-Q-T-Y-K-F-F-E |
| 650 | B20 | N-G-Y-E-N-P-Q-Y-K-F-F-E |
| 651 | B21 | N-G-Y-E-N-P-T-Q-K-F-F-E |
| 652 | B22 | N-G-Y-E-N-P-T-Y-Q-F-F-E |
| 653 | B23 | N-G-Y-E-N-P-T-Y-K-Q-F-E |
| 654 | B24 | N-G-Y-E-N-P-T-Y-K-F-Q-E |
| 655 | B25 | N-G-Y-E-N-P-T-Y-K-F-F-Q |
| 656 | B26 | A-G-Y-E-N-P-T-Y-K-F-F-E |
| 657 | B27 | C-G-Y-E-N-P-T-Y-K-F-F-E |
| 658 | B28 | A-A-A-A-A-A-A-A-A-A-A-A |
| 659 | B29 | H-H-H-H-H-H-H-H-H-H-H-H |
| 660 | B30 | A-A-A-H-H-H-H-H-H-A-A-A |
| 661 | C 1 | D-G-Y-E-N-P-T-Y-K-F-F-E |
| 662 | C 2 | E-G-Y-E-N-P-T-Y-K-F-F-E |
| 663 | C 3 | F-G-Y-E-N-P-T-Y-K-F-F-E |
| 664 | C 4 | G-G-Y-E-N-P-T-Y-K-F-F-E |
| 665 | C 5 | H-G-Y-E-N-P-T-Y-K-F-F-E |
| 666 | C 6 | I-G-Y-E-N-P-T-Y-K-F-F-E |
| 667 | C 7 | K-G-Y-E-N-P-T-Y-K-F-F-E |
| 668 | C 8 | L-G-Y-E-N-P-T-Y-K-F-F-E |
| 669 | C 9 | M-G-Y-E-N-P-T-Y-K-F-F-E |
| 670 | C10 | N-G-Y-E-N-P-T-Y-K-F-F-E |
| 671 | C11 | P-G-Y-E-N-P-T-Y-K-F-F-E |
| 672 | C12 | Q-G-Y-E-N-P-T-Y-K-F-F-E |
| 673 | C13 | R-G-Y-E-N-P-T-Y-K-F-F-E |
| 674 | C14 | S-G-Y-E-N-P-T-Y-K-F-F-E |
| 675 | C15 | T-G-Y-E-N-P-T-Y-K-F-F-E |
| 676 | C16 | V-G-Y-E-N-P-T-Y-K-F-F-E |
| 677 | C17 | W-G-Y-E-N-P-T-Y-K-F-F-E |
| 678 | C18 | Y-G-Y-E-N-P-T-Y-K-F-F-E |
| 679 | C19 | W-G-Y-E-N-P-T-Y-K-F-F-E |
| 680 | C20 | Y-G-Y-E-N-P-T-Y-K-F-F-E |
| 681 | C21 | N-A-Y-E-N-P-T-Y-K-F-F-E |
| 682 | C22 | N-C-Y-E-N-P-T-Y-K-F-F-E |
| 683 | C23 | N-D-Y-E-N-P-T-Y-K-F-F-E |
| 684 | C24 | N-E-Y-E-N-P-T-Y-K-F-F-E |
| 685 | C25 | N-F-Y-E-N-P-T-Y-K-F-F-E |
| 686 | C26 | N-G-Y-E-N-P-T-Y-K-F-F-E |
| 687 | C27 | N-H-Y-E-N-P-T-Y-K-F-F-E |
| 688 | C28 | N-I-Y-E-N-P-T-Y-K-F-F-E |
| 689 | C29 | N-K-Y-E-N-P-T-Y-K-F-F-E |
| 690 | C30 | A-A-A-H-H-H-H-H-H-A-A-A |
| 691 | D 1 | N-L-Y-E-N-P-T-Y-K-F-F-E |
| 692 | D 2 | N-M-Y-E-N-P-T-Y-K-F-F-E |
| 693 | D 3 | N-N-Y-E-N-P-T-Y-K-F-F-E |
| 694 | D 4 | N-P-Y-E-N-P-T-Y-K-F-F-E |
| 695 | D 5 | N-Q-Y-E-N-P-T-Y-K-F-F-E |
| 696 | D 6 | N-R-Y-E-N-P-T-Y-K-F-F-E |
| 697 | D 7 | N-S-Y-E-N-P-T-Y-K-F-F-E |
| 698 | D 8 | N-T-Y-E-N-P-T-Y-K-F-F-E |
| 699 | D 9 | N-V-Y-E-N-P-T-Y-K-F-F-E |
| 700 | D10 | N-W-Y-E-N-P-T-Y-K-F-F-E |
| 701 | D11 | N-Y-Y-E-N-P-T-Y-K-F-F-E |
| 702 | D12 | N-G-A-E-N-P-T-Y-K-F-F-E |
| 703 | D13 | N-G-C-E-N-P-T-Y-K-F-F-E |
| 704 | D14 | N-G-D-E-N-P-T-Y-K-F-F-E |
| 705 | D15 | N-G-E-E-N-P-T-Y-K-F-F-E |
| 706 | D16 | N-G-F-E-N-P-T-Y-K-F-F-E |
| 707 | D17 | N-G-G-E-N-P-T-Y-K-F-F-E |
| 708 | D18 | N-G-H-E-N-P-T-Y-K-F-F-E |
| 709 | D19 | N-G-I-E-N-P-T-Y-K-F-F-E |
| 710 | D20 | N-G-K-E-N-P-T-Y-K-F-F-E |
| 711 | D21 | N-G-L-E-N-P-T-Y-K-F-F-E |
| 712 | D22 | N-G-M-E-N-P-T-Y-K-F-F-E |
| 713 | D23 | N-G-N-E-N-P-T-Y-K-F-F-E |
| 714 | D24 | N-G-P-E-N-P-T-Y-K-F-F-E |
| 715 | D25 | N-G-Q-E-N-P-T-Y-K-F-F-E |
| 716 | D26 | N-G-R-E-N-P-T-Y-K-F-F-E |
| 717 | D27 | N-G-S-E-N-P-T-Y-K-F-F-E |
| 718 | D28 | N-G-T-E-N-P-T-Y-K-F-F-E |
| 719 | D29 | N-G-V-E-N-P-T-Y-K-F-F-E |
| 720 | D30 | N-G-W-E-N-P-T-Y-K-F-F-E |
| 721 | E 1 | A-A-A-H-H-H-H-H-H-A-A-A |
| 722 | E 2 | A-A-A-A-A-A-A-A-A-A-A-A |
| 723 | E 3 | N-G-Y-E-N-P-T-Y-K-F-F-E |
| 724 | E 4 | N-G-Y-A-N-P-T-Y-K-F-F-E |
| 725 | E 5 | N-G-Y-C-N-P-T-Y-K-F-F-E |
| 726 | E 6 | N-G-Y-D-N-P-T-Y-K-F-F-E |
| 727 | E 7 | N-G-Y-E-N-P-T-Y-K-F-F-E |
| 728 | E 8 | N-G-Y-F-N-P-T-Y-K-F-F-E |
| 729 | E 9 | N-G-Y-G-N-P-T-Y-K-F-F-E |
| 730 | E10 | N-G-Y-H-N-P-T-Y-K-F-F-E |
| 731 | E11 | N-G-Y-I-N-P-T-Y-K-F-F-E |
| 732 | E12 | N-G-Y-K-N-P-T-Y-K-F-F-E |
| 733 | E13 | N-G-Y-L-N-P-T-Y-K-F-F-E |
| 734 | E14 | N-G-Y-M-N-P-T-Y-K-F-F-E |
| 735 | E15 | N-G-Y-N-N-P-T-Y-K-F-F-E |
| 736 | E16 | N-G-Y-P-N-P-T-Y-K-F-F-E |
| 737 | E17 | N-G-Y-Q-N-P-T-Y-K-F-F-E |
| 738 | E18 | N-G-Y-R-N-P-T-Y-K-F-F-E |
| 739 | E19 | N-G-Y-S-N-P-T-Y-K-F-F-E |
| 740 | E20 | N-G-Y-T-N-P-T-Y-K-F-F-E |
| 741 | E21 | N-G-Y-V-N-P-T-Y-K-F-F-E |
| 742 | E22 | N-G-Y-W-N-P-T-Y-K-F-F-E |
| 743 | E23 | N-G-Y-Y-N-P-T-Y-K-F-F-E |
| 744 | E24 | N-G-Y-E-A-P-T-Y-K-F-F-E |
| 745 | E25 | N-G-Y-E-C-P-T-Y-K-F-F-E |
| 746 | E26 | N-G-Y-E-D-P-T-Y-K-F-F-E |
| 747 | E27 | N-G-Y-E-E-P-T-Y-K-F-F-E |
| 748 | E28 | N-G-Y-E-F-P-T-Y-K-F-F-E |
| 749 | E29 | N-G-Y-E-G-P-T-Y-K-F-F-E |
| 750 | E30 | N-G-Y-E-H-P-T-Y-K-F-F-E |
| 751 | F 1 | N-G-Y-E-I-P-T-Y-K-F-F-E |
| 752 | F 2 | N-G-Y-E-K-P-T-Y-K-F-F-E |
| 753 | F 3 | N-G-Y-E-L-P-T-Y-K-F-F-E |
| 754 | F 4 | N-G-Y-E-M-P-T-Y-K-F-F-E |
| 755 | F 5 | N-G-Y-E-N-P-T-Y-K-F-F-E |
| 756 | F 6 | N-G-Y-E-P-P-T-Y-K-F-F-E |
| 757 | F 7 | N-G-Y-E-Q-P-T-Y-K-F-F-E |
| 758 | F 8 | N-G-Y-E-R-P-T-Y-K-F-F-E |
| 759 | F 9 | N-G-Y-E-S-P-T-Y-K-F-F-E |
| 760 | F10 | N-G-Y-E-T-P-T-Y-K-F-F-E |
| 761 | F11 | N-G-Y-E-V-P-T-Y-K-F-F-E |
| 762 | F12 | N-G-Y-E-W-P-T-Y-K-F-F-E |
| 763 | F13 | N-G-Y-E-Y-P-T-Y-K-F-F-E |
| 764 | F14 | N-G-Y-E-N-A-T-Y-K-F-F-E |
| 765 | F15 | N-G-Y-E-N-C-T-Y-K-F-F-E |
| 766 | F16 | N-G-Y-E-N-D-T-Y-K-F-F-E |
| 767 | F17 | N-G-Y-E-N-E-T-Y-K-F-F-E |
| 768 | F18 | N-G-Y-E-N-F-T-Y-K-F-F-E |
| 769 | F19 | N-G-Y-E-N-G-T-Y-K-F-F-E |
| 770 | F20 | N-G-Y-E-N-H-T-Y-K-F-F-E |
| 771 | F21 | N-G-Y-E-N-I-T-Y-K-F-F-E |
| 772 | F22 | N-G-Y-E-N-K-T-Y-K-F-F-E |
| 773 | F23 | N-G-Y-E-N-L-T-Y-K-F-F-E |
| 774 | F24 | N-G-Y-E-N-M-T-Y-K-F-F-E |
| 775 | F25 | N-G-Y-E-N-N-T-Y-K-F-F-E |
| 776 | F26 | N-G-Y-E-N-P-T-Y-K-F-F-E |
| 777 | F27 | N-G-Y-E-N-Q-T-Y-K-F-F-E |
| 778 | F28 | N-G-Y-E-N-R-T-Y-K-F-F-E |
| 779 | F29 | N-G-Y-E-N-S-T-Y-K-F-F-E |
| 780 | F30 | N-G-Y-E-N-T-T-Y-K-F-F-E |
| 781 | G 1 | N-G-Y-E-N-V-T-Y-K-F-F-E |
| 782 | G 2 | N-G-Y-E-N-W-T-Y-K-F-F-E |
| 783 | G 3 | N-G-Y-E-N-Y-T-Y-K-F-F-E |
| 784 | G 4 | N-G-Y-E-N-P-A-Y-K-F-F-E |
| 785 | G 5 | N-G-Y-E-N-P-C-Y-K-F-F-E |
| 786 | G 6 | N-G-Y-E-N-P-D-Y-K-F-F-E |
| 787 | G 7 | N-G-Y-E-N-P-E-Y-K-F-F-E |
| 788 | G 8 | N-G-Y-E-N-P-F-Y-K-F-F-E |
| 789 | G 9 | N-G-Y-E-N-P-G-Y-K-F-F-E |
| 790 | G10 | N-G-Y-E-N-P-H-Y-K-F-F-E |
| 791 | G11 | N-G-Y-E-N-P-I-Y-K-F-F-E |
| 792 | G12 | N-G-Y-E-N-P-K-Y-K-F-F-E |
| 793 | G13 | N-G-Y-E-N-P-L-Y-K-F-F-E |
| 794 | G14 | N-G-Y-E-N-P-M-Y-K-F-F-E |
| 795 | G15 | N-G-Y-E-N-P-N-Y-K-F-F-E |
| 796 | G16 | N-G-Y-E-N-P-P-Y-K-F-F-E |
| 797 | G17 | N-G-Y-E-N-P-Q-Y-K-F-F-E |
| 798 | G18 | N-G-Y-E-N-P-R-Y-K-F-F-E |
| 799 | G19 | N-G-Y-E-N-P-S-Y-K-F-F-E |
| 800 | G20 | N-G-Y-E-N-P-T-Y-K-F-F-E |
| 801 | G21 | N-G-Y-E-N-P-V-Y-K-F-F-E |
| 802 | G22 | N-G-Y-E-N-P-W-Y-K-F-F-E |
| 803 | G23 | N-G-Y-E-N-P-Y-Y-K-F-F-E |
| 804 | G24 | N-G-Y-E-N-P-T-A-K-F-F-E |
| 805 | G25 | N-G-Y-E-N-P-T-C-K-F-F-E |
| 806 | G26 | N-G-Y-E-N-P-T-D-K-F-F-E |
| 807 | G27 | N-G-Y-E-N-P-T-E-K-F-F-E |
| 808 | G28 | N-G-Y-E-N-P-T-F-K-F-F-E |
| 809 | G29 | N-G-Y-E-N-P-T-G-K-F-F-E |
| 810 | G30 | N-G-Y-E-N-P-T-H-K-F-F-E |
| 811 | H 1 | N-G-Y-E-N-P-T-I-K-F-F-E |
| 812 | H 2 | N-G-Y-E-N-P-T-K-K-F-F-E |
| 813 | H 3 | N-G-Y-E-N-P-T-L-K-F-F-E |
| 814 | H 4 | N-G-Y-E-N-P-T-M-K-F-F-E |
| 815 | H 5 | N-G-Y-E-N-P-T-N-K-F-F-E |
| 816 | H 6 | N-G-Y-E-N-P-T-P-K-F-F-E |
| 817 | H 7 | N-G-Y-E-N-P-T-Q-K-F-F-E |
| 818 | H 8 | N-G-Y-E-N-P-T-R-K-F-F-E |
| 819 | H 9 | N-G-Y-E-N-P-T-S-K-F-F-E |
| 820 | H10 | N-G-Y-E-N-P-T-T-K-F-F-E |
| 821 | H11 | N-G-Y-E-N-P-T-V-K-F-F-E |
| 822 | H12 | N-G-Y-E-N-P-T-W-K-F-F-E |
| 823 | H13 | N-G-Y-E-N-P-T-Y-K-F-F-E |
| 824 | H14 | N-G-Y-E-N-P-T-Y-A-F-F-E |
| 825 | H15 | N-G-Y-E-N-P-T-Y-C-F-F-E |
| 826 | H16 | N-G-Y-E-N-P-T-Y-D-F-F-E |
| 827 | H17 | N-G-Y-E-N-P-T-Y-E-F-F-E |
| 828 | H18 | A-A-A-H-H-H-H-H-H-A-A-A |
| 829 | H19 | G-S-H-H-H-H-H-H-G-S-S-A |
| 830 | H20 | A-A-A-A-A-A-A-A-A-A-A-A |
| 831 | H21 | N-G-Y-E-N-P-T-Y-F-F-F-E |
| 832 | H22 | N-G-Y-E-N-P-T-Y-G-F-F-E |
| 833 | H23 | N-G-Y-E-N-P-T-Y-H-F-F-E |
| 834 | H24 | N-G-Y-E-N-P-T-Y-I-F-F-E |
| 835 | H25 | N-G-Y-E-N-P-T-Y-K-F-F-E |
| 836 | H26 | N-G-Y-E-N-P-T-Y-L-F-F-E |
| 837 | H27 | N-G-Y-E-N-P-T-Y-M-F-F-E |
| 838 | H28 | N-G-Y-E-N-P-T-Y-N-F-F-E |
| 839 | H29 | N-G-Y-E-N-P-T-Y-P-F-F-E |
| 840 | H30 | N-G-Y-E-N-P-T-Y-Q-F-F-E |
| 841 | I 1 | N-G-Y-E-N-P-T-Y-R-F-F-E |
| 842 | I 2 | N-G-Y-E-N-P-T-Y-S-F-F-E |
| 843 | I 3 | N-G-Y-E-N-P-T-Y-T-F-F-E |
| 844 | I 4 | N-G-Y-E-N-P-T-Y-V-F-F-E |
| 845 | I 5 | N-G-Y-E-N-P-T-Y-W-F-F-E |
| 846 | I 6 | N-G-Y-E-N-P-T-Y-Y-F-F-E |
| 847 | I 7 | N-G-Y-E-N-P-T-Y-K-A-F-E |
| 848 | I 8 | N-G-Y-E-N-P-T-Y-K-C-F-E |
| 849 | I 9 | N-G-Y-E-N-P-T-Y-K-D-F-E |
| 850 | I10 | N-G-Y-E-N-P-T-Y-K-E-F-E |
| 851 | I11 | N-G-Y-E-N-P-T-Y-K-F-F-E |
| 852 | I12 | N-G-Y-E-N-P-T-Y-K-G-F-E |
| 853 | I13 | N-G-Y-E-N-P-T-Y-K-H-F-E |
| 854 | I14 | N-G-Y-E-N-P-T-Y-K-I-F-E |
| 855 | I15 | N-G-Y-E-N-P-T-Y-K-K-F-E |
| 856 | I16 | N-G-Y-E-N-P-T-Y-K-L-F-E |
| 857 | I17 | N-G-Y-E-N-P-T-Y-K-M-F-E |
| 858 | I18 | N-G-Y-E-N-P-T-Y-K-N-F-E |
| 859 | I19 | N-G-Y-E-N-P-T-Y-K-P-F-E |
| 860 | I20 | N-G-Y-E-N-P-T-Y-K-Q-F-E |
| 861 | I21 | N-G-Y-E-N-P-T-Y-K-R-F-E |
| 862 | I22 | N-G-Y-E-N-P-T-Y-K-S-F-E |
| 863 | I23 | N-G-Y-E-N-P-T-Y-K-T-F-E |
| 864 | I24 | N-G-Y-E-N-P-T-Y-K-V-F-E |
| 865 | I25 | N-G-Y-E-N-P-T-Y-K-W-F-E |
| 866 | I26 | N-G-Y-E-N-P-T-Y-K-Y-F-E |
| 867 | I27 | N-G-Y-E-N-P-T-Y-K-F-A-E |
| 868 | I28 | N-G-Y-E-N-P-T-Y-K-F-C-E |
| 869 | I29 | N-G-Y-E-N-P-T-Y-K-F-D-E |
| 870 | I30 | N-G-Y-E-N-P-T-Y-K-F-E-E |
| 871 | J 1 | N-G-Y-E-N-P-T-Y-K-F-F-E |
| 872 | J 2 | N-G-Y-E-N-P-T-Y-K-F-G-E |
| 873 | J 3 | N-G-Y-E-N-P-T-Y-K-F-H-E |
| 875 | J 5 | N-G-Y-E-N-P-T-Y-K-F-K-E |
| 876 | J 6 | N-G-Y-E-N-P-T-Y-K-F-L-E |
| 877 | J 7 | N-G-Y-E-N-P-T-Y-K-F-M-E |
| 878 | J 8 | N-G-Y-E-N-P-T-Y-K-F-N-E |
| 880 | J10 | N-G-Y-E-N-P-T-Y-K-F-Q-E |
| 881 | J11 | N-G-Y-E-N-P-T-Y-K-F-R-E |
| 882 | J12 | N-G-Y-E-N-P-T-Y-K-F-S-E |
| 883 | J13 | N-G-Y-E-N-P-T-Y-K-F-T-E |
| 885 | J15 | N-G-Y-E-N-P-T-Y-K-F-W-E |
| 887 | J17 | N-G-Y-E-N-P-T-Y-K-F-F-A |
| 888 | J18 | N-G-Y-E-N-P-T-Y-K-F-F-C |
| 889 | J19 | N-G-Y-E-N-P-T-Y-K-F-F-D |
| 890 | J20 | N-G-Y-E-N-P-T-Y-K-F-F-E |
| 891 | J21 | A-A-A-H-H-H-H-H-H-A-A-A |
| 892 | J22 | A-A-A-A-A-A-A-A-A-A-A-A |
| 893 | J23 | N-G-Y-E-N-P-T-Y-K-F-F-F |
| 894 | J24 | N-G-Y-E-N-P-T-Y-K-F-F-G |
| 895 | J25 | N-G-Y-E-N-P-T-Y-K-F-F-H |
| 896 | J26 | N-G-Y-E-N-P-T-Y-K-F-F-I |
| 897 | J27 | N-G-Y-E-N-P-T-Y-K-F-F-K |
| 898 | J28 | N-G-Y-E-N-P-T-Y-K-F-F-L |
| 899 | J29 | N-G-Y-E-N-P-T-Y-K-F-F-M |
| 900 | J30 | N-G-Y-E-N-P-T-Y-K-F-F-N |
| 901 | K 1 | N-G-Y-E-N-P-T-Y-K-F-F-P |
| 902 | K 2 | N-G-Y-E-N-P-T-Y-K-F-F-Q |
| 904 | K 4 | N-G-Y-E-N-P-T-Y-K-F-F-S |
| 905 | K 5 | N-G-Y-E-N-P-T-Y-K-F-F-T |
| 908 | K 8 | N-G-Y-E-N-P-T-Y-K-F-F-Y |
